# Eosinophils in solid cancers: sentinels, predictors, and therapeutic allies

**DOI:** 10.1186/s13046-026-03688-5

**Published:** 2026-03-12

**Authors:** Marie Gilon, Delphine Boulet, Vincent Bours, Guy Jerusalem, Claire Josse, Christine Gennigens

**Affiliations:** 1 Laboratory of Medical Oncology, GIGA-Research, University Hospital of Liège, Liège, 4000 Belgium; 2 Department of Medical Oncology, University Hospital of Liège, Liège, 4000 Belgium; 3 Department of Human Genetics, University Hospital of Liège, Liège, 4000 Belgium

**Keywords:** Eosinophils, Cancer, Tumor microenvironment, Prognostic biomarker, Immunotherapy

## Abstract

Eosinophils are multifunctional granulocytes traditionally recognized for their roles in allergic responses and defense against parasitic infections. However, their involvement in cancer biology has emerged as an area of research interest in recent years. Epidemiological studies consistently suggest a protective role against tumor development, while in established malignancies, their prognostic significance varies considerably between cancer types. The most compelling evidence for clinical utility has emerged in cancer immunotherapy, where both baseline and treatment-induced eosinophil elevations consistently correlate with improved checkpoint inhibitor responses, enhanced survival outcomes, but also increased immune-related adverse events across multiple tumor types. Mechanistic studies reveal dual anti- and protumoral functions mediated through direct cytotoxic effects via granule proteins and indirect immune microenvironment modulation. This review synthesizes current knowledge on eosinophils in cancer biology and discusses the translational challenges that must be addressed to harness their clinical potential as biomarkers and therapeutic targets in oncology.

## Background

Eosinophils have long been recognized primarily for their role in allergic diseases and parasitic infections. However, in the past two decades, their involvement in cancer biology has attracted growing attention, revealing a complex relationship that spans from cancer prevention to treatment response prediction.

These multifaceted immune cells have emerged as intriguing players in oncology, supported by a growing body of evidence. Epidemiological studies have consistently demonstrated inverse associations between eosinophil levels and cancer risk, particularly for patients with colorectal malignancies. In established cancer, they have been reported as prognostic biomarkers across numerous solid tumors, although their significance varies considerably among studies. Most notably, in the era of cancer immunotherapy, eosinophils have gained recognition as reliable predictors of both treatment response and immune-related toxicity. However, while their potential as biomarkers is increasingly recognized, their role as active effectors in antitumor immunity remains uncertain.

This review synthesizes current knowledge on eosinophils in cancer, with the primary aim of providing clinicians with a clear understanding of their potential relevance in the field of oncology. We begin with a concise overview of eosinophil biology to contextualize their role within broader immune networks, followed by a systematic assessment of clinical evidence regarding their contributions in solid malignancies. Mechanistic insights from in vitro and murine models are then examined to provide a deeper understanding of how eosinophils may influence tumor development and progression. Finally, we critically examine the substantial inconsistencies observed across the literature and highlight the principal challenges that must be addressed to enable effective integration of eosinophil-related findings into clinical practice.

## Eosinophil biology and physiology

### Development, characteristics, and physiological niches

Eosinophils are immune cells characterized by a cytoplasm densely packed with granules. These granules are a hallmark feature of the cells and inspired Paul Ehrlich in 1879 to name them *eosinophils*, referring to their affinity for eosin dye [1]. Morphologically, eosinophils are larger than most other leukocytes (12–17 µm in diameter) and display a multilobed nucleus [2].

**Fig. 1:**
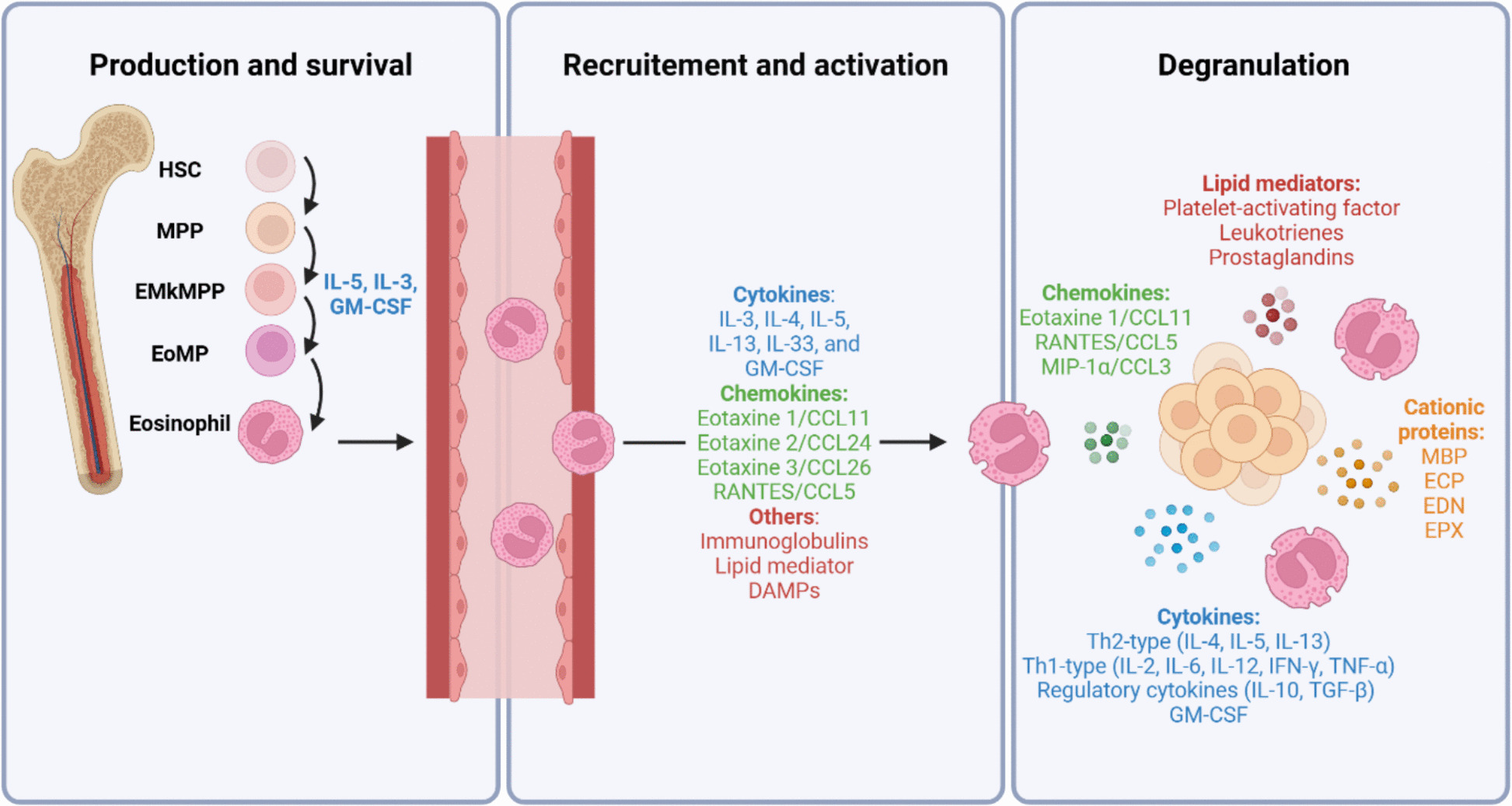
Overview of eosinophil biology and mediators. Eosinophils are produced in the bone marrow from erythroid-megakaryocyte-primed multipotent progenitors under the control of IL-5, IL-3, and GM-CSF. Following release into the circulation, they are recruited to tissues through chemokine gradients and activated by diverse mediators including cytokines, chemokines, and other mediators. Upon activation, eosinophils undergo degranulation, releasing cytotoxic cationic proteins, cytokines, chemokines, and lipid mediators

These myeloid-derived cells originate in the bone marrow from hematopoietic stem cells. Historically considered to stem directly from a common granulocyte–macrophage progenitor shared with neutrophils, recent research has shown that they arise from a lineage of progenitors expressing the GATA-1 transcription factor, which also give rise to mast cells, basophils, erythrocytes, and megakaryocytes, further differentiating into eosinophil-committed progenitors (Fig. 1) [3].

In the bloodstream, eosinophils represent less than 5% of total leukocytes and have a short half-life of 8 to 18 h [4, 5]. Under homeostatic conditions, they populate the thymus, secondary lymphoid tissues, gastrointestinal tract (excluding the esophagus), adipose tissue, lungs, and uterus [6, 7]. Once recruited into tissue, eosinophils can survive for up to two weeks [8].

Eosinophil production and survival are regulated primarily by interleukin-5 (IL-5), with additional contributions from IL-3 and granulocyte–macrophage colony-stimulating factor (GM-CSF). The recruitment and activation of eosinophils within tissues involve a complex network of mediators, including chemokines, cytokines, immunoglobulins (Ig), lipid mediators, and damage-associated molecular patterns (DAMPs). The key chemokines driving eosinophil migration are the C–C chemokine ligands CCL11, CCL24, and CCL26 (eotaxins 1, 2, and 3, respectively), as well as CCL5 (also known as RANTES), which predominantly signal through the C–C chemokine receptor type 3 (CCR3). Among the cytokines, IL-3, IL-4, IL-5, IL-13, IL-33, and GM-CSF play central roles in orchestrating eosinophil activity and tissue trafficking. Some of these mediators are produced by eosinophils themselves, exerting auto- and paracrine effects [9].

Upon activation, eosinophils undergo degranulation, releasing an extensive arsenal of preformed mediators that encompass cationic proteins, cytokines, chemokines, and lipid mediators, enabling rapid and versatile cellular responses (Table 1) [10, 11]. Cationic proteins are positively charged molecules that exhibit high affinity for negatively charged cell membranes and macromolecules, thereby conferring potent cytotoxic activity against parasites, bacteria, and malignant cells. Several of these proteins additionally possess ribonuclease activity, which endows them with antiviral properties [11].

**Table 1:** Factors released by eosinophils and their mechanisms of action

Category	Mediator	Mechanism of action	Ref
Cationic protein	Major basic protein (MBP)	Disrupts lipid bilayer membrane through surface charge interactions; induces membrane permeabilization and apoptosis	[10]
	Eosinophil cationic protein (ECP)	Induces pore formation in cell membranes; exhibits RNase activity with antiviral properties	[10]
	Eosinophil-derived neurotoxin (EDN)	Exhibits RNase activity with antiviral properties	[10]
	Eosinophil peroxidase (EPX)	Generates reactive oxygen species; induces oxidative damage	[10]
Cytokines	IL-4, IL-5, IL-13	Initiates Th2 inflammatory response	[12]
	IL-2, IL-6, IL-12, IFN-γ, TNF- α	Initiates Th1 inflammatory response	[12]
	IL-10, TGF- β	Exerts regulatory and immunosuppressive functions	[12]
	GM-CSF	Promotes leukocyte growth and differentiation; mediates autocrine eosinophil survival and activation	[13]
Chemokines	Eotaxin 1/CCL11	Mediates autocrine eosinophil recruitment and activation	[14]
	RANTES/CCL5	Regulates local inflammatory responses; recruits circulating leukocytes; mediates autocrine eosinophil activation	[14]
	MIP-1 α/CCL3	Regulates local inflammatory responses; recruits circulating leukocytes	[14]
Lipid mediators	Platelet-activating factor, Leukotrienes, Prostaglandins	Mediates inflammation; regulates leukocyte trafficking, endothelial adhesion, vascular permeability, smooth muscle contraction, and mucus secretion	[13]
Others	Charcot-Leyden crystal/galectin-10	Activates NLRP3 inflammasome; promotes neutrophilic inflammation, type 2 sensitization, and IgE synthesis	[9]
	Granzyme A and B, perforin	Induces cell apoptosis through proteolytic activity	[15, 16]

Although eosinophils are traditionally described as terminally differentiated cells upon bone marrow exit, accumulating evidence has demonstrated their functional plasticity, characterized by dynamic adaptation of transcriptional, phenotypic, and functional profiles in response to local microenvironmental cues. Distinct eosinophil subtypes have been identified across tissues and disease settings, including resident versus inflammatory eosinophils in the lung, basal versus activated eosinophils in the colon, and villus- versus crypt-associated eosinophils in the small intestine [17–19]. Moreover, transcriptional analyses have led to the characterization of type 1 and type 2 eosinophils, reflecting adaptation to T helper 1 (Th1)- or Th2-skewed inflammatory milieus [20]. This plasticity allows eosinophils to adapt their phenotype in response to their microenvironment and underlies their multifaceted roles in immunity, tissue remodeling and homeostasis.

### Physiological functions in immunity and tissue homeostasis

Eosinophils are predominantly recognized for their role in innate immunity, particularly in antiparasitic defense, while also demonstrating antibacterial, antiviral, and antifungal properties mediated through cytotoxic granule release. In addition to these innate functions, eosinophils have emerged as pivotal regulators of adaptive immunity through their capacity to activate and recruit immune cells via cytokine and chemokine secretion, as well as through antigen presentation capabilities [21, 22].

In healthy individuals, they also contribute to maintaining immune homeostasis across multiple organs. In the bone marrow, they promote plasma cell survival. In the gut, they sustain long-lived plasma cells, support IgA production, increase mucus protection, and regulate local immune responses, thereby preserving intestinal barrier integrity [23]. In the lungs, they regulate immune and tissue homeostasis, whereas thymic eosinophils contribute to central tolerance [17, 24].

Eosinophils are also involved in nonimmune physiological processes, including mammary gland development, tissue regeneration such as in the liver and skeletal muscle, and metabolic regulation, including glucose homeostasis and thermogenesis [25].

### Pathological roles across organ systems

Despite these protective roles, eosinophils also contribute significantly to the pathogenesis of various inflammatory and immune-mediated disorders. In allergic diseases, they are involved in the late-phase responses of IgE and T-cell–mediated hypersensitivity, amplifying inflammation through the release of proinflammatory mediators [9]. In the respiratory tract, eosinophils are central to asthma pathophysiology, contributing to mucus hypersecretion, epithelial damage, airway hyperresponsiveness, and structural remodeling, making anti-eosinophilic therapies pivotal in asthma treatment [6, 26]. Elevated eosinophil counts are also observed in subsets of patients with chronic obstructive pulmonary disease [27] as well as in those with chronic rhinosinusitis with nasal polyps [28].

Beyond respiratory pathologies, eosinophil-driven inflammation extends to diverse organ systems. In dermatological conditions, eosinophils contribute to atopic dermatitis and bullous pemphigoid [29, 30], whereas gastrointestinal involvement encompasses eosinophilic esophagitis, gastritis, colitis, and inflammatory bowel diseases [31, 32]. Eosinophils are further implicated in autoimmune conditions including eosinophilic myocarditis, eosinophilic granulomatosis with polyangiitis, neuromyelitis optica, and primary biliary cirrhosis [7]. Moreover, they mediate severe drug-induced hypersensitivity reactions, notably drug reaction with eosinophilia and systemic symptoms (DRESS) syndrome [33]. Eosinophils also constitute the principal effector cells in hypereosinophilic syndromes, a group of disorders characterized by persistent hypereosinophilia of diverse etiologies, associated with organ damage directly attributable to eosinophil-mediated toxicity [34].

This exploration of eosinophil biology highlights their functional diversity, encompassing both effector and regulatory roles in immunity, as well as contributions to tissue and metabolic homeostasis. The diversity of these functions stems from their intrinsic plasticity and the extensive variety of mediators within their signaling networks. The following section explores how this phenotypic and functional diversity confers both pro- and antitumoral roles to eosinophils, depending on the specific context.

## Eosinophils in hematological malignancies

Although this review concentrates on eosinophils biology within solid tumor microenvironments, the relationship between eosinophils and hematological cancers merits brief consideration, as it operates through markedly different mechanisms. Eosinophilia in hematological malignancies can arise via distinct pathways that fundamentally differ from the recruitment-based model observed in solid tumors.

First, eosinophilia in hematological malignancies can be reactive (or secondary) to the production of eosinophilopoietic cytokines, predominantly in lymphoid neoplasms, such as Hodgkin lymphoma, mature T-cell neoplasms, the lymphocytic variant of hypereosinophilic syndrome, and B- and T-cell lymphoblastic leukemia/lymphoma. In these settings, eosinophils represent primarily bystander cells responding to the cytokine milieu, though their role as active tumor microenvironment participants cannot be entirely excluded [35].

Second, eosinophilia that is associated with a hematological malignancy may also be neoplastic (or primary), derived from a malignant clone, usually in myeloid neoplasms (myeloid/lymphoid neoplasms with eosinophilia and tyrosine kinase gene fusions, acute myeloid leukemia with core binding factor translocations, mastocytosis, myeloproliferative neoplasms, myelodysplastic/myeloproliferative neoplasms, and myelodysplastic neoplasms). Here, eosinophils themselves harbor the oncogenic driver mutation, representing malignant rather than reactive cells [35].

However, eosinophils have also been descripted as prognostic markers in several hematological malignancies. In Hodgkin lymphoma, tissue eosinophilia represents a significant unfavorable prognostic factor, potentially through eosinophil-derived ligands (CD30) that promote Reed-Sternberg cell proliferation [36, 37]. Similarly, in adult T-cell leukemia/lymphoma, peripheral blood eosinophilia constitutes an independent adverse prognostic factor associated with reduced survival [38, 39]. Conversely, low eosinophil counts at diagnosis correlate with worse prognosis in multiple myeloma, suggesting a protective role in this setting [40]. These divergent prognostic associations underscore the context-dependent and disease-specific roles of eosinophils in hematological malignancies.

These hematological conditions are distinct conceptual framework requiring separate analysis from solid tumor biology, where eosinophils function as recruited immune effector cells within the tumor microenvironment, with potential direct cytotoxic or immunomodulatory activities. The pathophysiology, diagnostic implications, and therapeutic considerations of eosinophilia in hematological malignancies warrant comprehensive dedicated review and extend beyond the scope of this manuscript.

## Eosinophils in solid tumors

### Eosinophils and cancer risk

After evidence suggesting that allergies have a protective effect on the risk of several malignancies [41], researchers have focused their attention on eosinophils, as these cells are key players in allergic diseases and could mediate this association with cancer. Prizment et al*.* were the first to report in 2011 that higher blood eosinophil counts were associated with a reduced risk of colorectal cancer (CRC) in a prospective cohort of 10,675 cancer-free individuals followed for nearly 20 years. No significant association was observed between eosinophil levels and the risk of lung or breast cancer in this cohort [42]. Since then, multiple Mendelian randomization (MR) studies have corroborated these findings, further supporting a potential causal link between elevated eosinophil levels and reduced CRC risk [43–47].

In 2024, a large-scale prospective study of over 400,000 cancer-free individuals from the UK Biobank reported an inverse association between absolute eosinophil count (AEC) and overall cancer risk (hazard ratio (HR) = 0.97; 95% CI: 0.95–0.98). Across the 58 cancer types examined, eight showed borderline inverse associations (p < 0.05 but above the Bonferroni-adjusted threshold of p < 0.0009), including melanoma, cancers of the nose/middle ear, soft tissue/heart, gums/other oral sites, tongue, lung, colon, and breast. Conversely, a positive trend was observed for intrahepatic bile duct cancer [48]. These findings were later confirmed in the same cohort [49]. However, a separate population-based study of 359,950 individuals revealed no significant association between eosinophilia (defined as AEC > 500/mm^3^) and the subsequent risk of solid tumors [50].

Regarding specific cancer types, the protective effect of eosinophils on melanoma risk was supported by three MR studies [46, 47, 51]. Liu et al. reported a reduced hepatocellular carcinoma risk with increased eosinophil counts [52]. In contrast, lung cancer findings remain inconsistent: one MR analysis suggested that elevated eosinophils might increase risk in East Asian populations, whereas another did not confirm this observation [53, 54]. No association was found for prostate cancer [55].

Together, these findings support the hypothesis that eosinophils may play a protective role in tumor immunosurveillance, particularly in CRC.

### Eosinophils as prognostic biomarkers in solid tumors

In the context of cancer, eosinophils have been reported as a prognostic factor, with studies showing highly heterogeneous associations that are either favorable or unfavorable. It is essential to distinguish between studies evaluating circulating eosinophil counts and those assessing tumor-associated tissue eosinophilia (TATE), as their biological importance and prognostic implications may differ. In the following sections, we review the available evidence on the prognostic value of eosinophils across different solid malignancies (summarized in Table 2) and then discuss their specific role and clinical relevance in cancer immunotherapy separately.

**Table 2:** Reported prognostic and clinicopathological associations of eosinophils in solid tumors

Cancer type	Author	Eosinophil assessment	Biological correlation	Prognostic impact	Threshold (for circulating eosinophils)	Detection method (for TATE)
Glioma	Sharma [56]	Circulating eosinophils	Higher in gliomas vs benign tumors/controls		-	-
	Huang [57]	Circulating eosinophils	Negatively associated with grade (↓ eosinophils in high grades)		-	-
	Zhang [58]	Circulating eosinophils	Negatively associated with grade (↓ eosinophils in high grades)	Favorable for OS	80/mm^3^	-
	Madghuri [59]	Circulating eosinophils		Favorable for OS	150/mm^3^	-
	Zheng [60]	Circulating eosinophils		No association with outcome	-	-
	Zhong [61]	TATE		Favorable for OS	-	RNA-seq
Colorectal	Wei [62]	Circulating eosinophils		Favorable for DFS and OS	55/mm^3^ (DFS) and 95/mm^3^ (OS)	-
	Gao [63]	Circulating eosinophils		Favorable for OS	-	-
	Alsalman [64]	Circulating eosinophils		Unfavorable for DFS (left-sided CRC only)	0.2%	-
	Marinkovic [65]	Circulating eosinophils		Unfavorable for neoadjuvant RTCT response (rectal only)	-	-
	Prizment [66]	TATE	Negatively associated with stage (↓ eosinophils in high stages)	Favorable for CRC death risk (stromal eosinophils)	-	Anti-EPX
	Fernández-Aceñero [67]	TATE		Favorable for RFS and OS	-	H&E and Giemsa
	Nielsen [68]	TATE		Favorable for OS (submucosal eosinophils)	-	H&E
	Harbaum [69]	TATE	Negatively associated with stage and poor differentiation	Favorable for PFS and CSS (peritumoral eosinophils)	-	H&E
	Nagtegaal [70]	TATE		Favorable for recurrence rate and OS (peritumoral eosinophils)	-	Anti-EG-2
	Klintrup [71]	TATE		Favorable for OS (margin eosinophils)	-	H&E
	Ramadan [72]	TATE		Favorable for OS (peritumoral eosinophils)	-	H&E
	Baumann [73]	TATE		Favorable for PFS (margin eosinophils)	-	H&E
	Väyrynen [74]	TATE	Negatively associated with stage (↓ eosinophils in high stages)	Favorable for PFS and OS (peritumoral eosinophils)	-	H&E
	Cho [75]	TATE	Higher in carcinoma vs dysplasia		-	-
Esophageal	Li [76]	Circulating eosinophils		Favorable for PFS and OS	125/mm^3^	
	Yang [77]	Circulating eosinophils and TATE		Favorable for PFS and OS	2%	H&E
	Zhang [78]	TATE		Favorable for OS	-	H&E
	Ishibashi [79]	TATE	Negatively associated with vascular and LN invasion	Favorable for OS (LN metastasis group)	-	Luna
	Jacobse [80]	TATE	Negatively associated with stage (↓ eosinophils in high stages)		-	Anti-EPX
Gastric	Abu-Shawer [81]	Circulating eosinophils	Positively associated with metastatic disease at diagnosis		-	-
	Iwasaki [82]	TATE	Positively associated with poor differentiation and invasion depth	Favorable for OS	-	H&E
	Cuschieri [83]	TATE		Favorable for OS (stromal eosinophils)	-	H&E
Pancreatic	Ciesielski [84]	Circulating eosinophils		Favorable for OS	100/mm^3^	-
	Okhuma [85]	Circulating eosinophils		Favorable for OS	-	-
	Holub [86]	Circulating eosinophils (ELR)		Favorable for PFS and OS	0.04	-
Cholangio-carcinoma	Shinke [87]	Circulating eosinophils		Favorable for OS	356/mm^3^	-
Hepatocellular carcinoma	Liu [88]	Circulating eosinophils	Higher risk of microvascular invasion		-	-
	Steel [89]	Circulating eosinophils		Favorable for OS	-	-
	Shao [90]	Circulating eosinophils (NER)		Favorable for PFS	102	-
	Wang [91]	TATE		Favorable for OS	-	RNA-seq + Anti-EPX
Head and neck	Kaur [92]	Circulating + TATE	Positively associated with differentiation		-	H&E
	Li [93]	Circulating eosinophils		Favorable for PFS, DMFS and OS	50/mm^3^	-
	Ye [94]	Circulating eosinophils (NER)		Favorable for DMFS and OS	29.6	-
	Gopinathan [95]	TATE	Positively associated with differentiation		-	H&E
	Jain [96]	TATE	Higher in OSCC vs dysplasia		-	Congo red
	Jain [97]	TATE	Higher in nonmetastatic vs metastatic group		-	Carbol chromotrope
	Siddiqui [98]	TATE	Negatively associated with differentiation		-	H&E
	De Paz [99]	TATE	Positively associated with stage and tumor depth	Unfavorable for OS	-	H&E
	Lee [100]	TATE	Positively associated with LN metastasis and perineural/lymph vascular invasion	Unfavorable for OS	-	H&E
	Oliveira [101]	TATE	Positively associated with stage	No association with DFS and OS	-	H&E
	Peurala [102]	TATE		Favorable for OS	-	H&E
	Dorta [103]	TATE		Favorable for OS	-	H&E
	Thompson [104]	TATE		Favorable for OS	-	H&E
	Fuji [105]	TATE		Favorable for DFS and OS (EGFR-positive patients)	-	H&E
	Sassier [106]	TATE		No association with OS	-	H&E
	Ercan [107]	TATE		No association with OS	-	H&E
	Leighton [108]	TATE		No association with dPFS and OS	-	H&E
Lung	Wang [109]	Circulating eosinophils		Favorable for OS	-	-
	Alashkar [110]	Circulating eosinophils		Unfavorable for OS	500/mm^3^	-
	Sun [111]	TATE		Favorable for OS	-	RNA-seq
	Ye [112]	TATE	Positively associated with stage and LN metastasis	Unfavorable for OS	-	Anti-EPO
	Takeuchi [113]	Eosinophilic pleural effusion		Favorable for OS	-	-
Mesothelioma	Willems [114]	Circulating eosinophils		Unfavorable for PFS and OS	220/mm^3^	-
Renal	Gunduz [115]	Circulating eosinophils		Favorable for PFS	-	-
	Wang [116]	Circulating eosinophils		Unfavorable for PFS and OS	5%	-
	Li [117]	TATE		Favorable for OS	-	RNA-seq
	Pan [118]	TATE		Favorable for OS	-	RNA-seq
Bladder	Temiz [119]	Circulating eosinophils		Unfavorable for recurrence during BCG therapy	149.5/mm^3^ or 0.185%	-
	Nunez-Nateras [120]	TATE		Favorable for BCG response	-	Anti-EPX
	Zaffran [121]	TATE		No association with BCG response	-	Anti-EPX and RNA-seq
	Popov [122]	TATE		Favorable for recurrence	-	H&E
Penile	Ono [123]	TATE	Positively associated with stage		-	H&E
Testicular	Kalavska [124]	Circulating eosinophils		Unfavorable for PFS	-	-
Endometrial	Holub [125]	Circulating eosinophils (ELR)		Unfavorable for OS	-	-
Cervical	Holub [126]	Circulating eosinophils		Favorable for OS	280/mm^3^	-
	Chun [127]	Circulating eosinophils		Unfavorable for PFS	-	-
	Bethwaite [128]	TATE		Favorable for OS	-	Anti-MBP
	van Driel [129]	TATE	Positively associated with infiltration depth and tumor size	Unfavorable for OS	-	H&E
Breast	Onesti [130]	Circulating eosinophils		Favorable for TTF and BCSS	1.50%	-
	Ownby [131]	Circulating eosinophils		Favorable for TTR	55/mm^3^	-
	Cihan [132]	Circulating eosinophils		No association with PFS or OS	-	-
	Chouliaras [133]	TATE		Favorable for DFS	-	RNA-seq
	Okcu [134]	TATE		Favorable for OS (luminal A and B)	-	H&E
	Ali [135]	TATE		Favorable for PFS and OS	-	RNA-seq

#### Central nervous system tumors

Several retrospective studies have investigated the relationship between preoperative circulating eosinophil levels and glioma. Sharma et al. described significantly elevated eosinophil counts at diagnosis in glioma patients compared to those with benign brain tumors or healthy controls [56]. Two additional studies reported that eosinophil levels decreased with increasing glioma grade, being higher in low-grade than in high-grade tumors [57, 136].

Eosinophil levels have also been associated with prognosis in glioma patients. The ENS score, which combines the eosinophil count and neutrophil-to-lymphocyte ratio, has been developed as an independent prognostic marker correlated with both tumor grade and overall survival (OS) [58]. Prognostic thresholds have been identified, with more than 150 eosinophils/mm^3^ distinguishing survival groups and levels above 500 cells/mm^3^ conferring an even greater survival advantage [59]. Furthermore, analysis of 703 patients from The Cancer Genome Atlas (TCGA) demonstrated that intratumoral eosinophil infiltration was positively correlated with 5-year OS [61].

#### Digestive tract cancers

##### Colorectal cancer

CRC represents one of the most extensively studied cancer types in terms of eosinophil prognostic value, with analyses spanning peripheral blood and tumor microenvironment (TME) assessments. In two large cohorts of stage I–III CRC patients, including patients treated with adjuvant chemotherapy, higher pretreatment blood eosinophils were associated with improved survival; thresholds of 55/mm^3^ and 95/mm^3^ were proposed for predicting disease-free survival (DFS) and OS, respectively [62, 63]. In contrast, Alsalman et al*.* reported that elevated eosinophil counts (> 0.2%) were linked to shorter DFS, although this was significant only for left-sided CRC [64]. In stage III–IV rectal cancer patients receiving neoadjuvant chemoradiotherapy, Marinkovic et al*.* described that higher pretreatment eosinophil levels were associated with poorer treatment response [65].

Blood eosinophils may also help predict hypersensitivity reactions (HSRs) to oxaliplatin, a chemotherapy agent commonly used in adjuvant or metastatic CRC, as elevated counts are associated with a greater risk of developing HSRs during treatment [137–139].

Multiple lines of evidence support the favorable prognostic role of TATE in CRC. Eosinophil infiltration progressively decreases from low-grade dysplasia to adenocarcinoma, with reduced stromal eosinophil density observed in advanced tumor stages [74, 75]. TATE correlates with favorable histopathological characteristics, including the absence of lymphovascular invasion and reduced depth of tumor invasion [69, 70]. A comprehensive meta-analysis of 36 studies across multiple cancer types demonstrated that TATE was associated with improved OS in patients with solid tumors (HR = 0.82, 95% CI 0.68–0.99, *p =* 0.041), with this relationship being particularly strong in patients with CRC (HR = 0.70, 95% CI 0.58–0.84, *p =* 0.001) [140]. These findings are further supported by additional CRC-specific studies consistently linking TATE to favorable clinical outcomes [70–73].

Importantly, eosinophil localization within the TME appears to influence their prognostic impact. Studies have distinguished between different histological compartments, including the stromal, epithelial, and peritumoral regions. The majority of investigations have emphasized the importance of peritumoral eosinophils, whereas stromal eosinophils have demonstrated clinical relevance in fewer studies [42, 69–74].

Given that the Immunoscore—a validated prognostic tool for assessing CD3 + and CD8 + lymphocyte infiltration in stage I-III CRC—has been established to predict recurrence, these findings raise the intriguing possibility of incorporating eosinophil-related parameters to increase its prognostic precision. In support of this concept, Baumann's study demonstrated that while eosinophil infiltration was positively associated with survival, it showed a limited correlation with existing Immunoscore components, suggesting its independent prognostic value [73].

##### Esophageal cancer

In resectable esophageal squamous cell carcinoma, TATE has been associated with improved OS, particularly in patients with lymph node metastases [78, 79]. TATE also correlates with key pathological features such as vascular invasion, lymph node involvement, and recurrence [79]. Eosinophil density appears to be inversely related to cancer progression, with studies reporting decreased infiltration during the transition from Barrett’s esophagus to esophageal adenocarcinoma, and from early-stage to advanced-stage (≥ T3) tumors [141, 142].

In advanced esophageal squamous cell carcinoma patients treated with chemotherapy or radiochemotherapy, elevated pretreatment blood eosinophil counts and eosinophil-to-lymphocyte ratio (ELR) predicted improved survival outcomes [76, 77]. Moreover, both high tumor and blood eosinophil levels following treatment serve as favorable prognostic indicators [77].

Notably, eosinophilic esophagitis (EoE), an allergic disorder characterized by pathological eosinophil infiltration, does not appear to increase esophageal malignancy risk [143–145]. Murine models even suggest a protective effect where EoE inflammation significantly reduces esophageal tumor burden, indicating that EoE-associated epithelial remodeling may limit carcinogenesis [146].

##### Gastric cancer

In localized gastric cancer, high TATE levels have been associated with favorable prognosis [82, 83]. In contrast, elevated circulating eosinophil counts correlate with greater likelihood of metastatic disease at diagnosis [81]. Additionally, growing evidence suggests that eosinophils may contribute to the development of intestinal-type gastric metaplasia, a well-established precancerous lesion and risk factor for gastric adenocarcinoma [147].

##### Pancreatic cancer

In localized pancreatic cancer, low circulating eosinophil levels or ELR at diagnosis predict shorter OS [84–86]. However, one study reported no correlation between baseline eosinophils and either metastatic status or OS in a mixed cohort of localized and metastatic cases [148].

##### Cholangiocarcinoma

For cholangiocarcinoma, limited data exist, with only one study investigating the prognostic value of eosinophils. In a cohort of 81 patients, including 29 who received adjuvant chemotherapy, low postoperative eosinophil counts were associated with shorter OS [87].

##### Hepatocellular carcinoma

In hepatocellular carcinoma (HCC), higher peripheral eosinophil levels are associated with improved survival, whereas a lower neutrophil-to-eosinophil ratio (NER) is correlated with smaller tumor size and longer progression-free survival (PFS) [89, 90]. Conversely, Liu et al*.* demonstrated an association between elevated eosinophils and microvascular invasion, although not with recurrence [88]. Transcriptomic analyses from the TCGA and Gene Expression Omnibus (GEO) datasets revealed that higher eosinophil infiltration scores were correlated with longer survival and greater expression of immune checkpoint genes, with enrichment analyses suggesting links to T-cell activation, proliferation, and differentiation [91]. Immunohistochemistry of stage II HCC samples confirmed the presence of eosinophils within the tumors and adjacent connective tissue, with higher eosinophil peroxidase (EPX) expression in the tumor tissue than in its nontumor counterparts [91].

#### Head and neck cancer

In head and neck cancer (HNC), TATE has been extensively studied, though the clinical significance of circulating eosinophils remains largely poorly investigated beyond treatment-related studies. Indeed, one study reported a correlation between the baseline eosinophil count and degree of differentiation in oral lesions [92].

At the tissue level, eosinophil infiltration in oral squamous cell carcinoma (OSCC) shows distinct patterns: infiltration is greater in OSCC than in dysplastic tissue [96], more abundant in well-differentiated versus poorly differentiated tumors [92, 95], and greater in non-metastatic than in metastatic cases [96, 97].

Prognostic associations remain inconsistent. Increased eosinophil infiltration has been linked to improved OS in patients with OSCC [102, 103], laryngeal cancer [104], and epidermal growth factor receptor (EGFR)-positive nasopharyngeal carcinoma (NPC) [105]. Conversely, other studies reported adverse associations in OSCC [99] and mixed HNC cohorts [100], whereas some found no prognostic value in laryngeal cancer [106, 107] or in NPC [108]. In support of the adverse associations, TATE-enriched HNC samples were associated with aggressive features such as lymph node metastasis, perineural invasion, lymphovascular invasion and tumor budding [99–101, 149].

In NPC, the level of circulating eosinophils has also been shown to be predictive of treatment response. A lower NER is correlated with better survival outcomes [94], whereas higher baseline eosinophil counts (> 150/mm^3^) predict greater reduction in the lymph node volume following chemotherapy [150]. Importantly, eosinophil dynamics during treatment appear to be prognostically significant: patients experiencing eosinophil depletion had worse OS and PFS, whereas those maintaining elevated counts at the end of treatment had more favorable prognoses [93].

#### Lung cancer

In lung cancer, eosinophil findings remain conflicting. Whereas one study observed lower eosinophil infiltration in non-small cell lung cancer (NSCLC) tissue than in adjacent normal lung [151], another reported the opposite results in a smaller cohort [112].

Evidence for protective effects includes improved prognosis associated with increasing blood eosinophil counts during (chemo)radiotherapy treatment in stage III–IV NSCLC patients [109]. RNA sequencing data revealed positive correlations between intratumoral eosinophil infiltration and patient survival [111], whereas eosinophilic pleural effusion, defined as pleural fluid containing ≥ 10% eosinophils, was associated with prolonged OS [113].

Conversely, detrimental effects have been reported, such as significantly shorter survival in NSCLC patients with blood eosinophil counts > 500/mm^3^ [110]. Higher eosinophil infiltration has also been associated with shorter survival, advanced TNM stage, and increased lymph node metastasis [112]. Additionally, elevated eosinophil levels during docetaxel treatment are suspected to contribute to HSRs in both lung and breast cancers [152].

In mesothelioma, a single study reported that higher baseline eosinophils (≥ 220/mm^3^) were associated with shorter PFS and OS following treatment with pemetrexed combined with cisplatin or carboplatin [114].

#### Genitourinary cancers

In clear cell renal cell carcinoma (ccRCC), analyses of the GEO and TCGA databases reported associations between higher eosinophil infiltration and longer OS, although the overall eosinophil abundance was low, warranting cautious interpretation [117, 118].

In the context of tyrosine kinase inhibitor (TKI) therapy, two studies explored the predictive value of eosinophils. Wang et al*.* reported no association between the baseline eosinophil count and survival but observed that an increase in eosinophils > 5% two months after TKI initiation was correlated with improved OS and PFS [116]. Moreover, Gunduz et al*.* linked higher pretreatment eosinophils to better PFS [115].

In bladder cancer, the role of eosinophils remains controversial. In non–muscle-invasive bladder cancer patients treated with Bacillus Calmette-Guerin, some studies associated increased blood eosinophils with recurrence [119], whereas others reported increased tissue eosinophil infiltration and degranulation in responders [120] or even no significant difference [121]. In surgically treated patients, a higher stromal eosinophil density was observed in those with recurrence, although it did not impact DFS [122].

In penile cancer, extremely limited data exist. One small study (*n =* 17) revealed that eosinophil infiltration was greater in early- than in advanced-stage disease, with stromal infiltration in advanced-stage disease showing a nonsignificant trend toward improved survival (5-year: 60% vs 0%, *p =* 0.058) [123].

In prostate cancer, while one study reported that the ELR was associated with Gleason score upgrading, another revealed that the ELR did not predict ISUP score upgrading [153, 154]. Moreover, blood eosinophil counts do not differ between healthy individuals and prostate cancer patients, or between Gleason score groups [155, 156].

In testicular germ cell tumors, lower pretreatment eosinophil levels correlated with advanced/metastatic disease and shorter PFS in 84 chemotherapy-naïve patients [124].

#### Gynecological cancers

In cervical cancer, the prognostic relevance of eosinophils remains controversial. Holub et al*.* reported that higher blood eosinophil levels and ELR correlated with better OS in patients treated with radiotherapy, chemotherapy and/or surgery [126]. Conversely, Chun et al*.* descripted that a higher absolute eosinophil count at diagnosis predicted shorter PFS following surgical resection of intraepithelial neoplasia [127]. Furthermore, Bethwaite et al*.* observed that a higher intratumoral eosinophil density was associated with improved 5-year survival [128] whereas van Driel et al. reported the opposite, linking dense eosinophilic infiltrates to larger tumor size, deeper invasion, and poorer survival, independent of lymph node or margin status. Notably, in the latter study, blood eosinophil levels were not associated with outcome [129].

In endometrial cancer, an elevated pretreatment ELR and eosinophil-to-neutrophil-to-lymphocyte ratio (ENLR) were significantly associated with worse OS in a cohort of 163 patients receiving adjuvant (chemo-)radiotherapy [125].

#### Breast cancer

In breast carcinoma, circulating eosinophils have variable prognostic associations. In a retrospective study of 930 patients with stage I–III breast cancer treated with surgery, Onesti et al*.* observed that a higher baseline relative eosinophil count (REC) was significantly associated with improved time-to-treatment failure and breast cancer–specific survival. The eosinophil and lymphocyte product (ELP) also predicted better outcomes [130]. Similarly, Ownby et al*.* reported that a lower preoperative eosinophil level was associated with a significantly higher relapse rate [131]. In contrast, in a cohort of 350 patients treated with adjuvant chemoradiotherapy, Cihan et al*.* found no significant association between the number of circulating eosinophils and PFS or OS [132].

Regarding TATE, the results are equally mixed. In a TCGA cohort of 1069 patients, no significant association with OS was observed, although a trend toward improved DFS emerged (*p =* 0.06), with TATE linked to more immunogenic tumors and specific immune infiltrates [133]. TATE has also been correlated with aggressive features, including triple-negative status, a high Ki-67 index and low estrogen receptor (ER) expression, although the survival impact was restricted to luminal subtypes [134]. Finally, a study of RNAseq data from over 10,000 cases revealed that eosinophil-related signatures predicted improved outcomes in ER-positive but not in ER-negative tumors [135].

### Eosinophils in cancer immunotherapy

Immune checkpoint inhibitors (ICIs) have reshaped the treatment landscape of multiple cancers, especially melanoma, NSCLC and renal cell carcinoma (RCC). Despite their substantial clinical impact, only a subset of patients achieves durable benefit, while others exhibit limited efficacy or develop significant immune-related adverse events (IRAEs). This variability underscores the need for reliable biomarkers to better predict therapeutic response, optimize patient selection, and anticipate toxicity.

Among the candidates explored, eosinophils have gained attention as readily accessible and cost-effective markers. Baseline counts, early treatment-induced increases, and eosinophil-based ratios have all been associated with treatment response, improved survival and IRAEs development across various tumor types. This section synthesizes current evidence linking eosinophil dynamics to ICI efficacy and toxicity.

Baseline eosinophil counts are consistently associated with clinical outcomes in ICI-treated patients. Goldschmidt et al. conducted a pancancer study including over 18,000 patients and reported a positive correlation between baseline eosinophil counts and OS. Multiple subsequent studies have confirmed that higher pretreatment eosinophil levels are associated not only with improved OS, but also with increased response rates and prolonged PFS across various ICIs and tumor types, including melanoma [157–160], NSCLC [161–163], RCC [164, 165], hepatocellular carcinoma [166–168], urothelial carcinoma [169], and HNC [170, 171].

Early studies in melanoma patients receiving ICIs revealed that eosinophil counts often rise during therapy [172, 173]. These dynamic changes carry prognostic relevance, with favorable outcomes associated with early eosinophil rises, even within normal limits [172, 174–184] and to relative proportions exceeding 5% during therapy [163, 185–187]. Okauchi et al. further demonstrated a gradient effect, with eosinophil counts highest in patients who achieved complete responses, followed by those with partial responses, stable disease, and lowest in progressive disease [163].

However, eosinophil elevation does not invariably indicate a response. Simon et al*.* observed that while all ICI-responders presented increased eosinophil counts, nearly 50% of nonresponders did as well [174]. Similarly, Verhaart et al*.* reported that eosinophils increase in both responding and nonresponding patients, whereas Singh et al*.* described a case of marked hypereosinophilia (2860/mm^3^) under nivolumab despite disease relapse [188, 189].

Importantly, prognostic thresholds differ depending on the assessment timepoint: lower cut-offs (REC ≥ 1.5% or AEC ≥ 50–200/μL) are typically predictive at baseline, whereas higher thresholds (REC ≥ 5% or AEC ≥ 300/μL) are commonly reported during treatment (Table 3). Interestingly, one study suggested that eosinophil counts within the range of 100–500/mm^3^ were associated with improved outcomes under ICI therapy, whereas excessively high levels (> 500/mm^3^) correlated with poorer survival, suggesting the existence of an optimal therapeutic range [161].

**Table 3:** Eosinophil threshold values for immunotherapy outcomes: comparison across multiple studies

Author	Cancer type	Evaluation timepoint	Threshold suggested (/mm^3^)	Threshold suggested (%)	Outcome associated
Amman [159]	Melanoma	Baseline	130		PFS
Caliman [162]	NSCLC	Baseline	130		PFS and OS
Chen [166]	Hepatocellular carcinoma	Baseline	45		OS
Ferrucci [160]	Melanoma	Baseline		1.50%	OS
Martens [158]	Melanoma	Baseline	50		OS
Mehra [190]	Melanoma and NSCLC	Baseline	200		OS
Minohara [171]	Head and neck	Baseline		1.50%	PFS and OS
Mota [169]	Urothelial	Baseline	100		OS
Nishikawa [170]	Head and neck	Baseline	15		PFS and OS
Rossari [167]	Hepatocellular carcinoma	Baseline	70		PFS and OS
Toshida [168]	Hepatocellular carcinoma	Baseline	170		PFS
Weide [157]	Melanoma	Baseline		1.50%	OS
Zahoor [165]	Renal	Baseline	100		Progression risk
Alves [186]	NSCLC	During treatment		5%	PFS and OS
Chen [166]	Hepatocellular carcinoma	During treatment	105		PFS
Ghebeh [187]	Breast cancer	During treatment	300		PFS
Moreira [185]	Melanoma	During treatment		5%	OS
Okauchi [163]	NSCLC	During treatment	300	5%	PFS
Osawa [179]	NSCLC	During treatment	330	5%	TTF
Yoshimura [180]	Renal	During treatment	329		PFS and OS

Several studies have demonstrated that the prognostic value of eosinophils is usually specific to ICI-treated patients, with this association being absent in cohorts receiving alternative therapeutic regimens. In melanoma, REC was prognostic only in ipilimumab-treated patients (alone or with chemotherapy), but not in those receiving chemotherapy alone [160]. In RCC, baseline eosinophils showed prognostic value exclusively in ICI-treated patients, with no effect on those receiving TKIs [164]. Similarly, in NSCLC, no prognostic value was found in patients receiving radiochemotherapy, in contrast with the ICI-treated cohort, whereas in urothelial cancer, the predictive value was absent in chemotherapy-treated patients [169, 179].

Although eosinophils demonstrate consistent prognostic associations in the immunotherapy setting, they are unlikely to serve as standalone biomarkers in clinical practice. Instead, they may be optimally integrated into composite scores. Nishikawa et al*.* developed a prognostic score for HNC that combines baseline eosinophil counts, their changes after two weeks of ICI therapy, and performance status, successfully predicting OS [170]. Similarly, Beulque et al*.* demonstrated that incorporating eosinophils into the International Metastatic Renal Cell Carcinoma Database Consortium (IMDC) model improved its prognostic performance in RCC [164].

Eosinophils can also be integrated into ratios with other white blood cells to enhance their prognostic significance. The most extensively studied is NER. In a pancancer meta-analysis, elevated baseline NER was significantly associated with poorer OS; since a high NER reflects a relatively lower eosinophil count, this finding is consistent with previous observations. However, no physiological reference range has been established for NER, resulting in heterogeneous cut-offs across studies (ranging from 14 to 102), which are often derived from ROC curve analyses or cohort medians [191]. Furthermore, lower NER values during treatment have also been correlated with improved outcomes [164, 192, 193].

In addition to their prognostic role, both baseline and on-treatment eosinophil counts have been consistently associated with increased IRAE risk across multiple studies [194–197]. Given that IRAE occurrence has been suggested to be correlated with improved survival under ICI therapy, these observations could further strengthen the predictive significance of eosinophils [198–200]. Although not formally included in clinical guidelines, several case reports have shown that anti-IL-5 monoclonal antibodies (mepolizumab and benralizumab) have achieved complete resolution of eosinophil-related IRAEs. Dupilumab, an antibody targeting IL-4 and IL-13—key cytokines produced by eosinophils—is recommended by major oncology societies for managing cutaneous IRAEs [201]. These observations underscore the role of eosinophils not only as biomarkers but also as likely effectors in IRAE development.

Collectively, these findings establish eosinophils—whether evaluated at baseline, monitored dynamically during therapy, or incorporated into composite scores—as robust but complex biomarkers in immunotherapy. Their consistent associations with both treatment efficacy and toxicity, combined with their accessibility and cost-effectiveness, position them as valuable tools for optimizing ICI therapy, although likely in combination with other biomarkers rather than as isolated predictors.

### Hypereosinophilia as paraneoplastic syndrome

Paraneoplastic hypereosinophilia is an uncommon but clinically significant phenomenon observed in solid tumors. It is defined as a persistent elevation of peripheral blood eosinophil counts > 1500/mm^3^ in the absence of secondary causes such as infection or allergy. The underlying mechanism is generally attributed to aberrant secretion of eosinophilopoietic cytokines by tumor cells, including IL-5, IL-3 and GM-CSF [202].

Clinically, paraneoplastic hypereosinophilia has been described in multiple cancer types, such as thyroid [203], renal cell [204], lung [205], pancreatic [206], gallbladder [207], ovarian [208], and bladder carcinoma [209]. In many cases, eosinophil counts normalize after surgical resection [210] or systemic treatment [211, 212], while recurrence of eosinophilia following resection may herald tumor relapse [202]. Importantly, paraneoplastic hypereosinophilia is often associated with poor prognosis [202].

Clinically, it may remain asymptomatic [211] or manifest with allergic-like symptoms [210], respiratory complaints such as dyspnea [208], or even organ damage [213, 214]. Thus, paraneoplastic hypereosinophilia represents both a potential biomarker of tumor activity and a clinically relevant complication that may require careful monitoring.

## Mechanistic insights: fundamental perspectives on eosinophils

### Eosinophils in tumor immune surveillance

The concept of immune surveillance, first proposed by Burnet and Thomas in 1957, suggests that the immune system continuously monitors and eliminates emerging malignant cells to prevent tumor development [215]. Within this framework, eosinophils are considered potential effector cells contributing to early tumor control. Despite epidemiological data linking eosinophil levels and cancer, the mechanistic basis of this relationship lacks robust evidence. Studies have therefore explored how eosinophils may either limit early malignant transformation or, alternatively, promote tumor initiation.

As they are key players in mucosal homeostasis, eosinophil deficiency alters the mucosal immune microenvironment in mice [216]. By supporting intestinal barrier integrity, eosinophils help prevent pathogen invasion, epithelial injury, and dysbiosis, all of which drive chronic inflammation and, ultimately, carcinogenesis. These mechanisms provide a rationale for the observed associations between allergies, eosinophil activation, and reduced CRC risk.

Furthermore, several studies suggest that eosinophils can restrain malignant progression from precancerous stages. In murine models, eosinophil depletion facilitates the evolution from dysplasia to invasive carcinoma, whereas their presence limits tumor outgrowth. This protective effect is driven by CCL11 expression in epithelial cells in precancerous lesions and is directly mediated by eosinophils [80, 217, 218].

However, eosinophils may also exert protumorigenic effects under specific circumstances. In gastric pathology models, they promote the transition from chronic gastritis to intestinal metaplasia, a preneoplastic lesion [219]. Similarly, in carcinogen-induced hamster OSCC models, eosinophil depletion reduces the tumor burden and delays tumor onset [220], whereas other studies reported their involvement in cervical cancer progression [221] and tongue squamous cell carcinoma development [222]. These findings highlight the dual role of eosinophils, which act either as guardians in immune surveillance or as facilitators of tumor-promoting inflammation.

### Eosinophils in antitumor immunity

Several murine studies have demonstrated that altering eosinophil levels, either through genetic models or manipulation of key cytokine pathways, directly influences tumor growth and metastatic dissemination. In breast cancer, elevated eosinophil counts reduce pulmonary metastases, whereas deficiency enhances spread [223]. Similarly, IL-5 deficiency or blockade increased lung metastasis in melanoma, while IL-33 administration delayed both primary tumor growth and metastasis [224, 225]. In CRC, IL-33 also restricts tumor progression by increasing eosinophil infiltration [226]. In these studies, the effects of IL-5 and IL-33 were confirmed to depend on eosinophils. However, in an ovarian cancer model in which IL-33 delayed tumor progression, eosinophil depletion reduced but did not completely abolish the antitumor effect of IL-33, suggesting the involvement of CD4^+^ Th2 cells and tumor-associated macrophages [227].

Eosinophils have been shown to exert direct cytotoxic effects on various tumor cell lines in numerous in vitro studies [228]. In tissue, these cells accumulate predominantly at the host–tumor interface, where they establish close and sustained contact with tumor cells [229]. This intimate contact is mediated by the interaction between the integrin LFA-1, a heterodimer composed of CD11a and CD18 expressed on eosinophils, and ICAM-1 on tumor cells, forming an immunological synapse [230]. This process enables polarized degranulation, through which eosinophils release a variety of proapoptotic cytotoxic mediators, including major basic protein (MBP), eosinophil cationic protein (ECP), eosinophil-derived neurotoxin (EDN), reactive oxygen species, granzymes A and B, and TNF-α [15, 16, 80, 231, 232]. These factors act synergistically to induce apoptosis and selectively eliminate tumor cells. Beyond degranulation, eosinophils can also trigger Fas/FasL-dependent apoptosis in cancer cells [233]. More recently, eosinophil extracellular trap cell death (ETosis), a nonapoptotic cell death process, has been identified as another mechanism of tumor control [234]. Eosinophils also promote vascular normalization and downregulate epithelial-to-mesenchymal transition pathways, therefore reducing metastatic potential [235, 236].

**Fig. 2:**
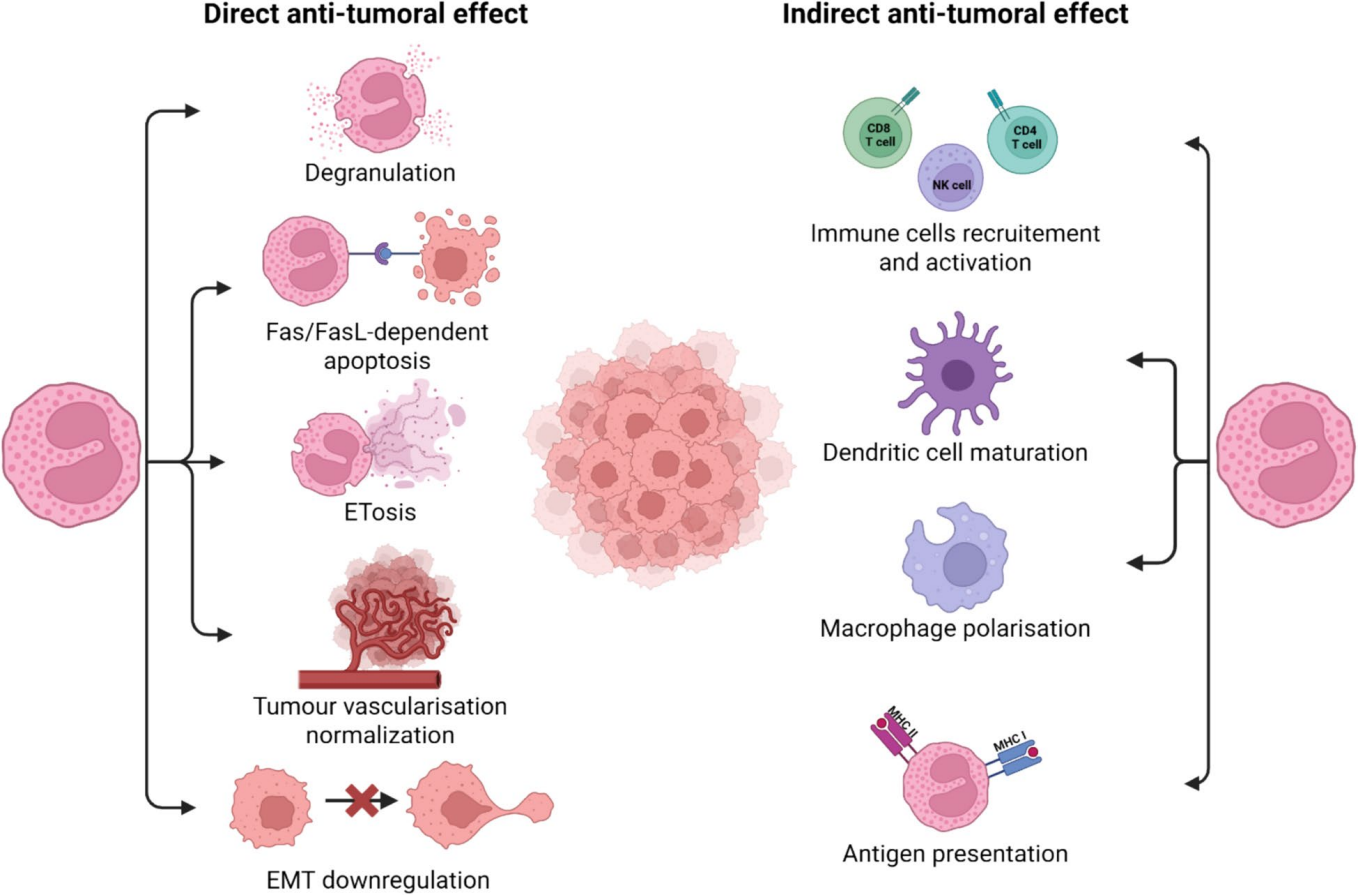
Direct and indirect antitumoral effects of eosinophils

Eosinophils function as crucial modulators of the immune TME through multiple complementary mechanisms. They actively recruit and activate cytotoxic effector cells, including CD8 + and CD4 + T lymphocytes as well as NK cells [225, 237–239], while promoting dendritic cell maturation to enhance antigen presentation [240]. Eosinophils can reprogram tumor-associated macrophages toward an antitumoral M1 phenotype while suppressing protumorigenic SPP1 + macrophages, thereby reshaping the myeloid compartment [235, 241]. Although their antigen-presenting capacity remains incompletely characterized in the tumor context, this function likely contributes to their immunomodulatory effects [242] (Fig. 2, Table 4).

**Table 4:** Summary of eosinophil anti-tumor mechanisms described across different cancer types

Cancer type	Mechanism	Ref
Esophageal cancer	- Direct cytotoxicity through reactive oxygen species secretion - Suppression of IL-17	[80]
Gastric cancer	- Direct cytotoxicity, notably through ETosis	[229, 234]
Colorectal cancer	- Direct cytotoxicity, notably through the secretion of ECP, EDN, TNF-alpha, granzyme A - Inhibition of macrophage differentiation to protumorigenic SPP1 + cells	[16, 217, 226, 230]
Hepatocarcinoma	- Direct cytotoxicity through TNF-alpha secretion	
Fibrosarcoma	- Direct cytotoxicity	[218]
Breast cancer	- Direct cytotoxicity through EPX secretion - CD4 + and CD8 + T-cells recruitment - NK cells recruitment	[223, 238, 239]
Melanoma	- Recruitment and activation of CD8 + T cells and NK cells - Polarization of macrophages to M-1 phenotype - Vascularization normalization - Direct cytotoxicity - Inhibition of tumor growth	[225, 235–237]
Prostate	- Direct cytotoxicity through MBP secretion	[231]
Glioblastoma	- Direct cytotoxicity through Fas/FasL interaction	[233]

Beside well-documented antitumor properties, eosinophils can also exert protumoral functions. In a breast cancer model, IL-33 promoted metastatic dissemination; however, this effect was pleiotropic and only partially dependent on eosinophils [243]. Mechanistically, eosinophils may facilitate metastasis by enhancing tumor cell recruitment through CCL6–CCR1 signaling [244]. They contribute to the establishment of an immunosuppressive TME by stimulating suppressive myeloid cell expansion in the bone marrow via IL-4 secretion, attracting regulatory T cells through CCL22 release, and promoting macrophage polarization toward a protumoral M2 phenotype [245–247]. Additionally, EPX exerts protumorigenic effects by activating HER2 signaling to stimulate tumor cell proliferation and by remodeling the tumor stroma through angiogenesis induction, collagen deposition, and myofibroblast proliferation [248, 249]. Finally, Charcot–Leyden crystals derived from eosinophil galectin-10 protein have been reported to protect tumor cells from chemotherapy-induced apoptosis [250].

The apparently paradoxical roles of eosinophils in cancer are increasingly understood as context-dependent, largely determined by their activation state and specific signals present within the TME. Naïve eosinophils are generally inert, whereas activated eosinophils acquire effector functions. Unactivated eosinophils displayed no cytotoxic properties in vitro, whereas appropriate stimulation markedly increased precancerous cells apoptosis [80]. Similarly, eosinophils from allergic donors, primed by chronic immune activation, exhibited enhanced tumoricidal capacity compared to those from nonallergic individuals, which is mediated by increased CD11a/CD18 adhesion molecule expression [230, 251]. Thus, the functional status of eosinophils appears to outweigh their simple presence within the TME, and the dual role of eosinophils could be related to different phenotypes. However, while distinct tissue-resident and recruited populations can be identified in inflammatory diseases such as asthma [17], no major transcriptional or proteomic differences were detected between Siglec-F^int^ and Siglec-F^hi^ eosinophils in metastatic lungs [238]. Similarly, the classification of hypodense versus normodense eosinophils does not explain eosinophil duality, as both show cytotoxic activity [251].

Although IL-5 and IL-33 are both central regulators of eosinophil activation, accumulating evidence suggests that they drive distinct functional phenotypes. Notably, IL-33–stimulated eosinophils appear more cytotoxic to tumor cells compared to IL-5–stimulated counterparts. Andreone et al*.* showed that IL-33 enhances eosinophil migration, tumor cell adhesion, and granule convergence (suggesting degranulation), whereas IL-5 induces weaker adhesion and little or no degranulation [252]. Similarly, Gambardella et al*.* reported that IL-33–activated eosinophils release more extracellular vesicles (EVs) than do IL-5-stimulated ones, with transcriptomic analyses revealing that IL-33–derived EVs are enriched in tumor suppressor genes and epithelial regulation pathways, whereas IL-5–induced EVs display a distinct profile [236]. Nevertheless, IL-5 remains an important activator, as it enhances eosinophil cytotoxicity in vitro [80, 251]. Together, these findings suggest that while both IL-5 and IL-33 promote antitumor activity, IL-33 may prime eosinophils toward a more potent cytotoxic phenotype, whereas IL-5 contributes to their effector functions through complementary but mechanistically different pathways.

Beyond IL-5 and IL-33, multiple TME-derived factors regulate eosinophil recruitment and function. Eotaxins are considered the main chemokines involved in eosinophil trafficking. Tumor cells can directly secrete CCL11 and CCL24, but other TME components such as macrophages, cancer-associated fibroblasts, eosinophils themselves, and even mucosal-associated invariant T-cells can also contribute to their production [228, 253–255]. CCL11 levels are negatively regulated by autotaxin and dipeptidyl peptidase 4 (DPP4); inhibition of these enzymes increases eosinophil infiltration and reduces tumor growth in vivo [256, 257]. Nevertheless, eosinophil recruitment in tumors appears to rely only partially on the CCR3–CCL11/CCL24 axis, suggesting the involvement of additional chemoattractants [80, 238].

Among these, GM-CSF, which is secreted by cancer cells, has been shown to promote eosinophil recruitment and antitumor activity through activation of the interferon regulatory factor IRF5, an effect that is counteracted by IL-10 [233, 237]. Eosinophils are also responsive to DAMPs such as high-mobility group box 1 (HMGB1), which are released by necrotic tumor cells and stimulate their migration and activation [240, 258]. Other TME signals further influence eosinophil functions: IL-18 enhances eosinophil adhesion and cytotoxicity [230], whereas estrogens suppress their survival and cytotoxic potential via ERα signaling [259]. In addition, while TNF-α and IFN-γ do not directly increase eosinophil cytotoxicity, they stimulate eosinophils to secrete chemokines that recruit CD4⁺ and CD8⁺ T cells, thereby amplifying antitumor immunity [238]. Thymic stromal lymphopoietin (TSLP), which is secreted by tumor cells under hypoxic stress, has been linked to a protumoral eosinophil recruitment in cervical cancer [221].

Moreover, the impact of eosinophils in cancer may differ according to disease stage: several studies reported a limited influence on primary tumor growth but a role in metastatic spread, suggesting the potential importance of eosinophils in premetastatic niches formation [238, 241].

**Fig. 3:**
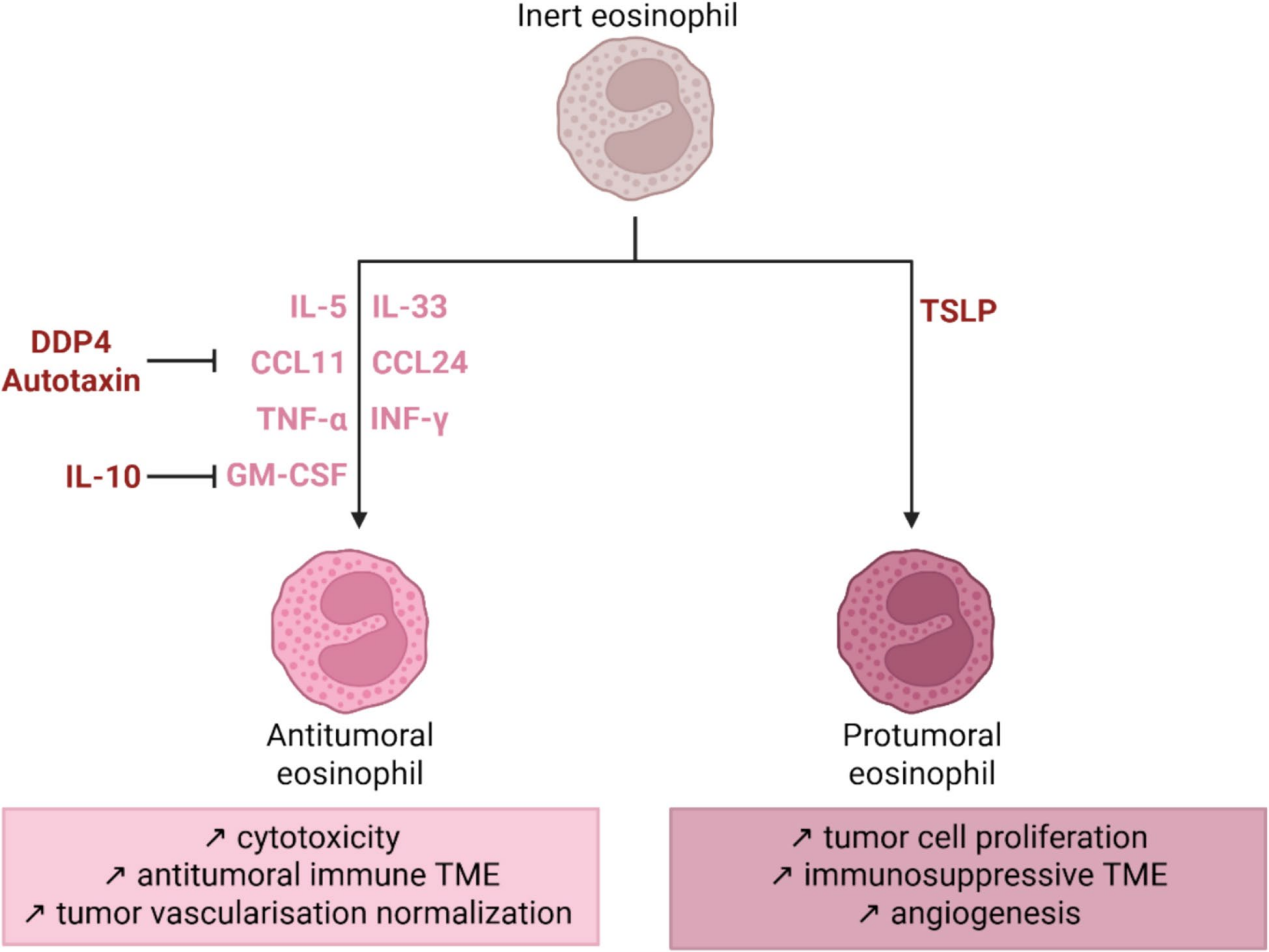
Context-dependent activation and dual functions of eosinophils in the TME

Overall, these observations indicate that eosinophil activity in tumors depends not only on their presence but on the specific combination of local activating signals. Their recruitment and functional polarization result from a complex interplay of cytokines, chemokines, and microenvironmental cues, ultimately determining whether they exert antitumor effects or promote tumor progression (Fig. 3).

### Eosinophils and immune checkpoint inhibitors

Tumor cells evade immune destruction by expressing ligands such as PD-L1 or CD80/CD86, which suppress T-cell activity through the engagement of inhibitory receptors. These receptors, notably cytotoxic T-lymphocyte antigen-4 (CTLA-4) and programmed cell death protein-1 (PD-1), normally function to prevent autoimmunity by inducing T-cell exhaustion and restricting proliferation. ICIs counteract this strategy by blocking CTLA-4, PD-1, or their ligands, thereby restoring T-cell activation and enabling effective tumor-specific immune responses [260]. Given the growing evidence of T-cell‒eosinophil cooperation in antitumor immunity, assessing their contribution to ICI efficacy is relevant.

In a preclinical breast cancer model, Blomberg et al*.* demonstrated that ICI plus cisplatine promoted IL-5 secretion by CD4⁺ T cells, which increased eosinophilopoiesis. Eosinophils were subsequently recruited into the TME via IL-33 and enhanced CD8⁺ T cell activation. Strikingly, eosinophil depletion abolished the therapeutic effect of combined ICI and cisplatin, whereas recombinant IL-33 administration improved the efficacy of ICI therapy by increasing eosinophil recruitment [261]. Moreover, Zheng et al*.* reported that anti-CTLA-4 therapy enhanced eosinophil infiltration in ICI-sensitive breast tumors (EO771, MMTV-PyVT) but not in resistant ones (MCaP0008). Activated CD4⁺ and CD8⁺ T cells promoted this recruitment via the secretion of chemokines (CCL5, CCL11) and cytokines (IL-5, IFN-γ). Eosinophil accumulation correlated with vascular normalization, characterized by reduced vascular density, improved perfusion, and enhanced pericyte coverage. Improved vascular normalization, in turn, facilitated CD8⁺ T cell infiltration, creating a positive feedback loop that reinforced antitumor immunity [262].

At the clinical level, transcriptomic analyses further support an active role of eosinophils in the ICI response. Using CIBERSORT deconvolution, Beulque et al*.* reported that eosinophil-high tumors were enriched in interferon signaling, PD-1 signaling, MHC-II antigen presentation, and costimulatory pathways, features typically associated with favorable ICI outcomes. In contrast, eosinophil-low tumors displayed TGF-β pathways, MYC targets, and other signatures linked to ICI resistance and tumor aggressiveness [164].

Taken together, these findings argue that eosinophils play an active role in shaping the efficacy of ICIs, rather than acting as mere biomarkers of response.

## Discussion

The comprehensive body of evidence presented in this review highlights the complex and multifaceted relationship between eosinophils and cancer, revealing both promising clinical applications and significant knowledge gaps that warrant careful consideration.

### The paradox of eosinophil dispensability

Despite the numerous functions attributed to eosinophils in maintaining physiological health, ranging from antimicrobial defense to tissue remodeling and immune regulation, mounting evidence suggests that these cells may not be essential for survival under normal conditions. The development of genetically modified eosinophil-deficient mice has demonstrated that these animals develop normally, reproduce successfully, and exhibit no overt pathological phenotypes under standard laboratory conditions. This finding is further supported by pharmacovigilance data from anti-eosinophilic biological therapies, particularly mepolizumab and benralizumab in severe asthma, which report excellent safety profiles from sustained eosinophil depletion in humans [263].

This apparent dispensability presents an evolutionary paradox: eosinophils are phylogenetically ancient cells, present across all vertebrate species and emerged early during vertebrate evolution. Their structural and functional conservation throughout evolutionary history, including the preservation of characteristic granule proteins and key regulatory pathways, contrasts with their apparent nonessential role in modern settings [264]. This paradox might be explained by the evolution of the immune system, which has become increasingly complex with the emergence and refinement of other immune cells that now largely compensate for eosinophil functions. Furthermore, the presumed redundancy of eosinophils in laboratory settings may not reflect their true physiological importance, as laboratory conditions typically lack the complex environmental stresses, pathogen challenges, and immune pressures that characterize natural ecosystems. Similarly, eosinophils may have been essential for ancestral survival in pathogen-rich environments, particularly against helminth infections that were prevalent throughout human evolutionary history, but their functions have become largely redundant in contemporary hygiene and medical settings.

### Eosinophil plasticity: the key to understanding their role in cancer

The difficulty in delineating the precise role of eosinophils in cancer largely stems from the fact that they have historically been investigated as a single, homogeneous population. However, accumulating evidence challenges this oversimplified view. As highlighted throughout this review, eosinophils display remarkable phenotypic plasticity, enabling them to dynamically adapt their functional programs in response to local microenvironmental cues.

This heterogeneity is observed across multiple contexts: distinct eosinophil phenotypes have been described based on their tissue of residence, with transcriptionally and functionally specialized subsets identified, notably in the gastrointestinal tract, lung, adipose tissue, synovium, and thymus. In eosinophil-associated disorders, multiple subpopulations with distinct roles have also been characterized. For example, in asthma, tissue-resident eosinophils exhibit an immunoregulatory phenotype, inhibiting dendritic cell maturation and limiting Th2 sensitization, whereas inflammatory eosinophils display a classical effector profile with enhanced pro-inflammatory gene expression [17].

The concept of eosinophil plasticity is further supported by extensive in vitro evidence demonstrating that eosinophils can acquire distinct functional phenotypes depending on environmental signals. For instance, Dolitzky et al*.* described type 1 and type 2 eosinophils, corresponding to anti-inflammatory and inflammatory phenotypes respectively, depending on the surrounding cytokine milieu [265]. As discussed above, the activation status of eosinophils and the signals driving their activation critically shape their cytotoxic potential, with IL-33–stimulated eosinophils exhibiting greater cytotoxicity toward tumor cells than IL-5–stimulated counterparts. Further evidence illustrating eosinophil plasticity has been comprehensively reviewed in [266].

Taken together, these findings provide a strong conceptual basis to postulate that eosinophils may adopt either pro- or antitumoral phenotypes depending on the tumor microenvironment, analogous to the well-established M1/M2 polarization of macrophages and N1/N2 phenotypes of neutrophils. Therefore, eosinophils should no longer be regarded as a uniform cell population but rather as a spectrum of functionally specialized states whose impact on tumor progression is context dependent.

As these putative subpopulations are discriminated by differences in surface marker expression, activation status, cytokine secretion profiles, and transcriptomic signatures, their identification necessitates the use of high-dimensional and high-precision technologies. Moreover, the field remains hindered by the absence of a unified classification framework. To date, no clear consensus has emerged defining robust eosinophil subsets, and substantial heterogeneity persists in the markers and methodologies employed across studies. These limitations represent a major barrier to a deeper comprehension of the role of eosinophil subpopulations in cancer.

In this context, improved characterization of distinct eosinophil subtypes and their associated functions within the tumor microenvironment is critical to better understand and ultimately harness the roles of eosinophils in cancer.

### Cancer prevention: epidemiological evidence versus mechanistic understanding

The inverse association between eosinophils and cancer risk, which is particularly evident in CRC through both epidemiological studies and MR analyses, represents one of the most robust findings in this field. However, this epidemiological strength is paradoxically matched by mechanistic weakness. While the involvement of eosinophils in mucosal immunity provides a plausible biological rationale—given that many cancers showing protective associations are mucosal or epithelial in origin—the precise mechanisms underlying this effect remain poorly characterized.

Importantly, eosinophil-deficient mouse models do not spontaneously develop tumors [263], suggesting that either the protective mechanisms are species specific, require specific environmental triggers absent under laboratory conditions, or that large cohorts of mice maintained over extended periods would be necessary to detect spontaneous tumor development. Moreover, there is currently no evidence that eosinophil deficiency—particularly in asthmatic patients treated with biologic therapies targeting IL-5 or its receptor—increases cancer risk, even if longer-term follow-up of these individuals is needed to rule out any potential late-onset effects [267]. This discrepancy between human epidemiological data and experimental models represents a significant challenge in translating these observations into therapeutic interventions.

### The challenge of prognostic heterogeneity

The prognostic value of eosinophils in established cancer presents a landscape of remarkable heterogeneity, with studies reporting favorable, unfavorable, or neutral associations even within the same tumor type. This variability likely stems from multiple interconnected factors that reflect both the biological complexity of eosinophils and the methodological challenges in studying them.

From a methodological standpoint, the lack of standardized approaches for eosinophil assessment represents a major source of variability. Differences in cut-off thresholds (see Table 2) and in the immunohistochemical markers used introduce substantial heterogeneity across studies. With regard to TATE, the distinction between stromal, epithelial, and peritumoral eosinophils, each potentially having different biological significance, represents an additional variable that is inconsistently addressed across studies. These methodological variations may explain why similar patient populations can yield conflicting results regarding the prognostic value of eosinophils.

Beyond methodological considerations, biological interactions between tumors and eosinophils further complicate prognostic interpretation. The description of paraneoplastic hypereosinophilia strongly suggests a bidirectional crosstalk between tumors and eosinophils, whereby tumor-derived cytokines drive eosinophil expansion and recruitment, while eosinophils in turn may influence tumor progression and immune responses. This observation adds an additional layer of complexity to the interpretation of circulating eosinophils: although generally regarded as protective immune effectors, markedly elevated eosinophil counts (> 1500/mm^3^) often reflect the presence of highly aggressive tumors and correlate with poor outcomes. Therefore, it is important for future studies assessing eosinophil prognostic value to account for this phenomenon and to consider excluding patients with paraneoplastic hypereosinophilia from analyses to avoid confounding effects. However, this approach raises a significant methodological challenge. While excluding patients with overt paraneoplastic hypereosinophilia (markedly elevated counts > 1500/mm^3^) may be straightforward, distinguishing early-stage paraneoplastic eosinophilia from truly protective eosinophil responses in the intermediate range remains problematic. In patients with moderately elevated eosinophil counts (e.g., 500–1500/mm^3^), it may be impossible to determine whether the elevation reflects an emerging paraneoplastic syndrome associated with aggressive tumor biology or a beneficial antitumor immune response. This ambiguity poses a risk of diluting the observed protective effect of eosinophils: if early paraneoplastic cases (associated with poor prognosis) are inadvertently grouped with truly protective eosinophilia (associated with good prognosis), the resulting mixed signal may mask the genuine prognostic benefit of eosinophil infiltration.

### The question of effector function

While eosinophils have been proposed as potential prognostic biomarkers in many cancer types, their role as active effectors in tumor biology remains uncertain. Experimental studies have attributed various antitumoral mechanisms to eosinophils, including direct cytotoxicity through granule proteins, immune cell activation, and vascularization normalization. However, these mechanistic insights are derived primarily from in vitro studies or preclinical animal models, with insufficient evidence from human studies to establish causality.

The current evidence is inadequate to support therapeutic interventions aimed at modulating eosinophil numbers or function in cancer patients. The risk‒benefit profile of such interventions remains unclear, particularly given the potential for unexpected consequences in patients with complex oncological conditions. Until a more robust mechanistic understanding is achieved, eosinophils should be viewed primarily as potential biomarkers rather than therapeutic targets in oncology.

### Immunotherapy: promising biomarkers with mechanistic gaps

In contrast to their uncertain role in cancer biology, eosinophil levels demonstrate a consistent association with both treatment response and IRAEs across multiple tumor types in the context of immune checkpoint inhibitors. The abundance of data on eosinophils in the context of ICIs, together with the use of anti-eosinophil drugs for managing IRAEs, suggests a mechanistic link between eosinophils and checkpoint inhibitor activity. However, the underlying biology of eosinophil involvement in ICI therapy remains poorly understood. Whether eosinophils serve merely as bystanders reflecting broader immune activation or function as active effectors in antitumor immunity remains to be definitively established. This mechanistic uncertainty limits our ability to optimize their clinical utility and raises important questions about the potential for therapeutic manipulation.

### Perspectives and challenges

The clinical implications of eosinophils in oncology encompass several key areas. Current evidence on eosinophils supports close monitoring of patients receiving anti-eosinophil therapies, as well as considering broader use of these therapies for the management of IRAEs. Prognostically, the integration of eosinophil parameters into composite scoring systems could enhance risk stratification. From a translational perspective, critical priorities include standardizing eosinophil assessment through consistent thresholds and reproducible TATE evaluation methods, while advancing mechanistic understanding through experimental modulation of eosinophil levels during ICI therapy.

## Conclusions

Eosinophils represent a fascinating example of cells with extensive functional potential but uncertain clinical necessity. In the context of cancer, they serve as potential biomarkers, particularly in immunotherapy, but their role as active participants in tumor biology remains to be fully elucidated. The heterogeneous nature of their prognostic significance across different cancers likely reflects their phenotypic plasticity and the methodological challenges in studying them consistently.

Moving forward, the field would benefit from standardized approaches to eosinophil assessment, development of validated composite biomarker scores, and mechanistic studies that bridge the gap between clinical observations and biological understanding. Only through such comprehensive investigations can the full potential of eosinophils in cancer medicine be realized to fully harness their potential in clinical settings.

## Data Availability

No datasets were generated or analyzed during the current study.

## Abbreviations

IL: Interleukin
GM-CSF: Granulocyte-macrophage colony-stimulating factor
Ig: Immunoglobulin
DAMP: Damage-associated molecular pattern
CCL: C-C chemokine ligands
CCR: C-C chemokine receptor
Th: T helper
HSC: Hematopoietic stem cell
MPP: Multipotent progenitor
EMkMPP: Erythroid–megakaryocyte-primed multipotent progenitor
EoMP: Eosinophil-mast cell progenitor
MIP-1-α: Macrophage inflammatory protein-1 alpha
MBP: Major basic protein
ECP: Eosinophil cationic protein
EDN: Eosinophil-derived neurotoxin
EPX: Eosinophil peroxidase
IFN‐γ: Interferon-gamma
TNF-α: Tumor necrosis factor alpha
TGF-β: Transforming growth factor beta
CRC: Colorectal cancer
MR: Mendelian randomization
AEC: Absolute eosinophil count
HR: Hazard ratio
TATE: Tumor-associated tissue eosinophilia
OS: Overall survival
TCGA: The Cancer Genome Atlas
TME: Tumor microenvironment
DFS: Disease-free survival
HSR: Hypersensitivity reaction
ELR: Eosinophil-to-lymphocyte ratio
EoE: Eosinophilic esophagitis
HCC: Hepatocellular carcinoma
NER: Neutrophil-to-eosinophil
PFS: Progression-free survival
GEO: Gene Expression Omnibus
HNC: Head and neck cancers
OSCC: Oral squamous cell carcinoma
EGFR: Epidermal Growth Factor Receptor
NPC: Nasopharyngeal carcinoma
NSCLC: Non-small cell lung cancer
ccRCC: Clear cell renal cell carcinoma
TKI: Tyrosine kinase inhibitor
ENLR: Eosinophil-to-neutrophil-to-lymphocyte ratio
REC: Relative eosinophil count
ELP: Eosinophil and lymphocyte percentages
ER: Estrogen receptor
RNA-seq: RNA sequencing
RTCT: Radiochemotherapy
RFS: Recurrence-free survival
CSS: Cancer-specific survival
H&E: Hematoxylin and eosin
LN: Lymph node
DMFS: Distant metastasis-free survival
dPFS: Distant progression-free survival
BCG: Bacillus Calmette-Guérin
TTF: Time-to-treatment failure
BCSS: Breast cancer-specific survival
TTR: Time to recurrence
ICI: Immune checkpoint inhibitor
RCC: Renal cell carcinoma
IRAE: Immune-related adverse event
IMDC: International Metastatic Renal Cell Carcinoma Database Consortium
ROS: Reactive oxygen species
EV: Extracellular vesicle
HMGB1: High–Mobility Group Box 1
TSLP: Thymic stromal lymphopoietin
CTLA-4: Cytotoxic T-lymphocyte antigen-4
PD-1: Programmed cell death protein-1
